# Supplementary material for: Specificity of Key Sex Determination Genes in a Mammal with Ovotestes: The European Mole Talpa europaea
Source: Animals (Basel). 2024 Jul 26;14(15):2180. doi: 10.3390/ani14152180 (PMC11311037; doi:10.3390/ani14152180)
Supplement: Supplementary file 1 [file animals-14-02180-s001.zip › animals-3012228-supplementary.pdf]

Supplementary materials

Specificity of Key Sex Determination Genes in a Mammal with Ovotestes: The European Mole *Talpa europaea*

Alexey Bogdanov [1], Maria Sokolova [1,2] and Irina Bakloushinskaya [1]

[1] Koltzov Institute of Developmental Biology, Russian Academy of Sciences, 119334 Moscow, Russia;
bogdalst@yahoo.com (A.B.); mmariasokol@gmail.com (M.S.)
[2] Biological Department, Lomonosov State University, 119234 Moscow, Russia
* Correspondence: i.bakloushinskaya@idbras.ru

Table S1. Data on European moles used in this work.

Locality	Coordinates	Voucher	Sex	Karyotype	GenBank accession numbers			
					Rspo1	*Eif2s3x*	*Eif2s3y*	*Sry*
1. Russia, Moscow region, Kashirsky district, Bolshoye Kropotovo village	N 54.8589, E 38.3578	26268	♀	—	PP841024<br>PP841031<br>PP841038	PP841012	—	—
2. Russia, Ivanovo region, Teykovsky district, 1 km east of Pershino village	N 56.89505, E 40.53591	26906	♀	—	—	PP841013	—	—
3. Russia, Ivanovo region, Teykovsky district, 3 km west of Pershino village	N 56.88764, E 40.46706	26948	♀	—	PP841025<br>PP841032<br>PP841039	PP841014	—	—
4. Russia, Ivanovo region, Teykovsky district, 4.4 km northeast of Pershino village, the right bank of Ukhtokhma River	N 56.92015, E 40.57665	27078	♀	—	PP841026<br>PP841033<br>PP841040	PP841015	—	—
5. Russia, Ivanovo region, Teykovsky district, 4.2 km northeast of Pershino village, the right bank of Ukhtokhma River	N 56.91361, E 40.57806	T23-15	♀	—	PP841027<br>PP841034<br>PP841041	PP841016	—	—
6. Russia, Ivanovo region, Teykovsky district, 1.5 km northwest of Obolsunovo village, the left bank of Ukhtokhma River	N 56.91389, E 40.59778	T23-16	♂	2n=34 (32 autosomes, X, and dot-like Y)	PP841028<br>PP841035<br>PP841042	PP841017	PP841020	PP841022
	N 56.91389, E 40.59778	T23-17	♀	2n=34 (32 autosomes and XX)	PP841029<br>PP841036<br>PP841043	PP841018	—	—
	N 56.91389, E 40.59889	T23-18	♂	—	PP841030<br>PP841037<br>PP841044	PP841019	PP841021	PP841023

Note: for the *Rspo1* gene, GenBank accession numbers, which are done in the upper, intermediate, and bottom lines, correspond to its initial, intermediate, and final fragments.

Table S2. Additional material from the GenBank.

Species	Gene	GenBank accession number and used fragment of the whole genome contig	Genome fragment description	Reference
Talpa occidentalis	*Rspo1*	RCFO01000009 (22721000–22736200)	Whole genome sequence	[33]
		XM_037499227	Predicted *Rspo1* mRNA	
Condylura cristata		XM_004678811	Predicted *Rspo1* mRNA	
Suncus etruscus		XM_049775528	Predicted *Rspo1* mRNA	
Sorex araneus		XM_004614239	Predicted *Rspo1* mRNA	
Sorex fumeus		XM_056123613	Predicted *Rspo1* mRNA	
Sorex palustris		JAOXYA010000032 (21064000–21081000)	Whole genome sequence	Duckett and Pirro, unpublished
Talpa occidentalis	*Eif2s3x*	RCFO01000015 (76145000–76165200)	Whole genome sequence	[33]
		XM_037512601	Predicted *Eif2s3* mRNA	
Mus musculus		NM_012010	mRNA	
Talpa occidentalis	*Eif2s3y*	RCFO01000018 (556000–598500)	Whole genome sequence	[33]
		XM_037518325	Predicted eukaryotic translation initiation factor 2 subunit 3, X-linked-like, mRNA	
Mus musculus		NM_012011	mRNA	
Talpa occidentalis	*Sry*	RCFO01000018 (1401100–1405550)	Whole genome sequence	[33]
		XM_037518332	Predicted *Sry* mRNA	
		X90843	The *Sry* gene fragment	[39]
Talpa europaea		X95595		
Talpa romana		X90863		
Neomys anomalus		X90864		
Crocidura suaveolens		X90865		
Erinaceus algirus		X90866		

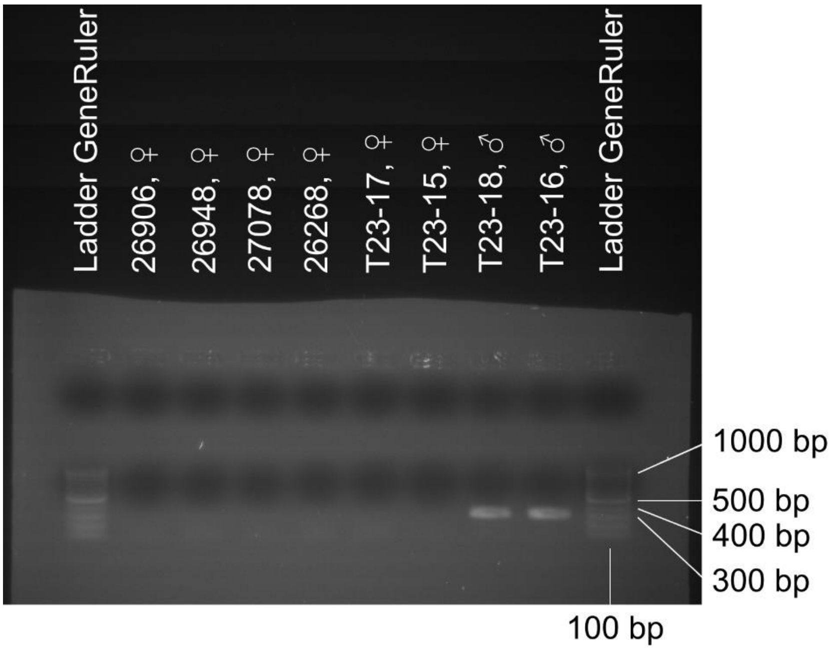

Figure S1. The pattern of 1% agarose gel electrophoresis of PCR products (≈60 ng) obtained by amplification of the *Eif2s3y* gene fragment in studied European moles; their vouchers see in Table 1. GeneRuler 100 bp DNA Ladder (Thermo Scientific) was used as a marker of fragment molecular lengths.

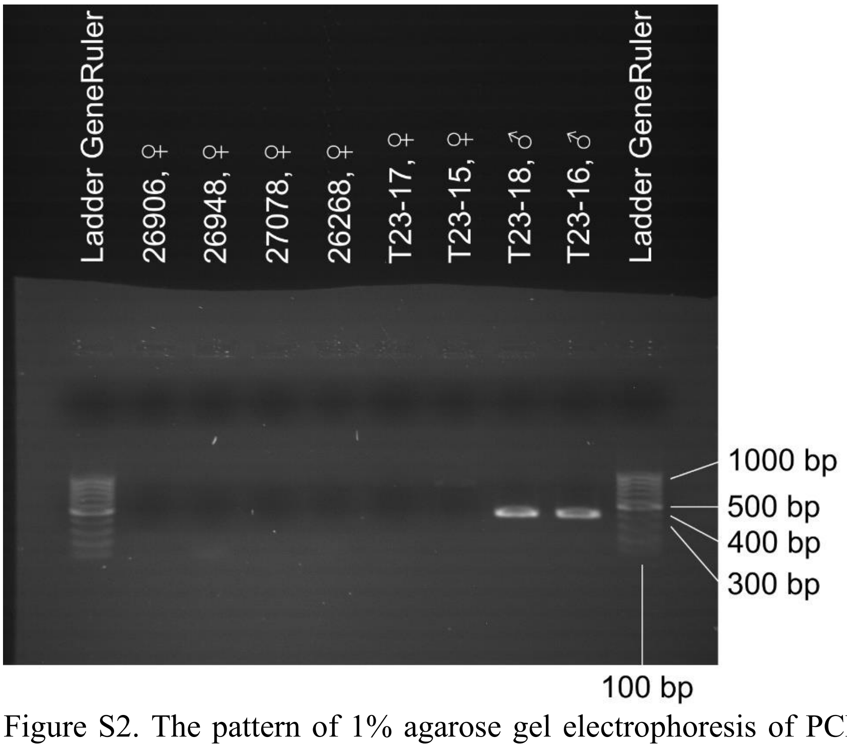

Figure S2. The pattern of 1% agarose gel electrophoresis of PCR products (≈60 ng) obtained by amplification of the *Sry* gene fragment with primers Sry-TFint and Sry-TRint in studied European moles; their vouchers see in Table 1. GeneRuler 100 bp DNA Ladder (Thermo Scientific) was used as a marker of fragment molecular lengths.

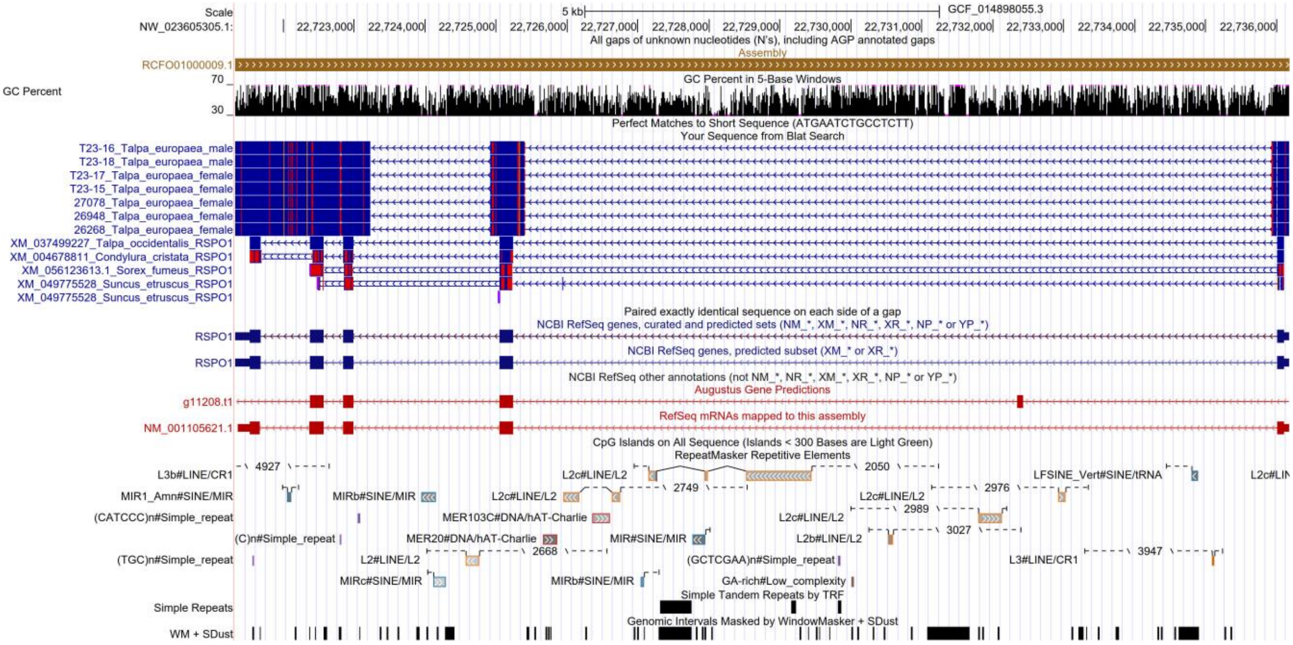

Figure S3. UCSC browser [37] screenshot of a 13 Mb window of the Iberian mole genome, highlighting the *Rspo1* gene structure in *T. europaea*.

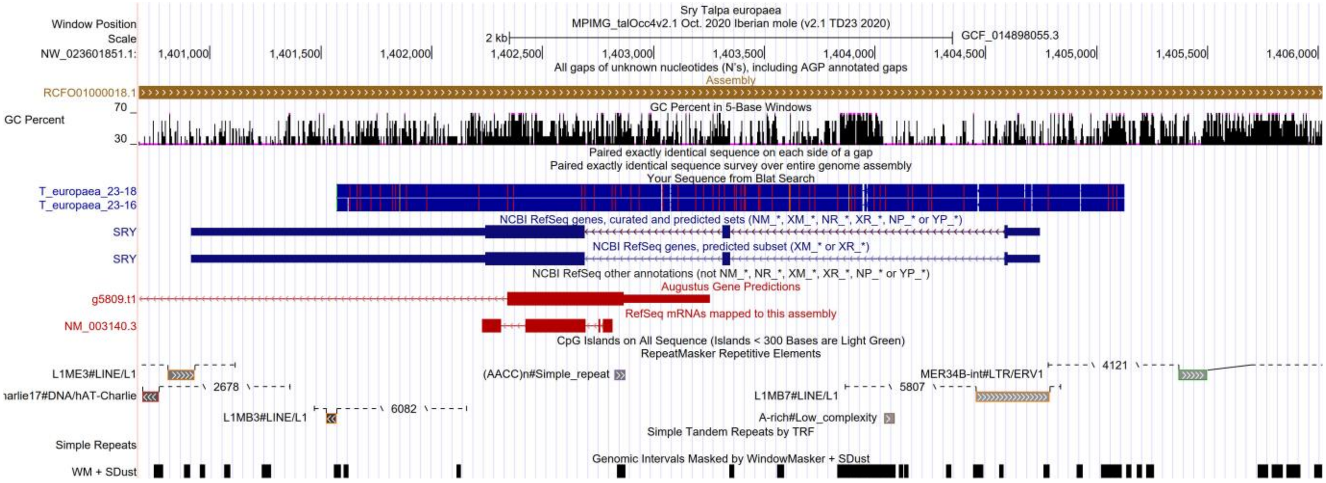

Figure S4. UCSC browser [37] screenshot of a 5 Mb window of the Iberian mole genome, highlighting the *Sry* gene structure in *T. europaea*.

Alignment S1,a. Alignment of nucleotide sequences of the *Rspo1* gene (the initial fragment I) of *T. occidentalis* and *T. europaea* specimens. The exon area is underlined in the upper sequence.

```
RCFO01000009_T.occidentalis GCAAGAGCCCGGCGCCCCCGTCCACCAGAGACCCCCTCCTCCCAGGGGCCTGTTGCTGGCGAGTGACTATGCGGC [ 75]
26268_T.europaea            ............................................................A.............. [ 75]
26948_T.europaea            ............................................................A.............. [ 75]
27078_T.europaea            ............................................................A.............. [ 75]
T23-15_T.europaea           ............................................................A.............. [ 75]
T23-16_T.europaea           ............................................................A.............. [ 75]
T23-17_T.europaea           ............................................................A.............. [ 75]
T23-18_T.europaea           ............................................................A.............. [ 75]

RCFO01000009_T.occidentalis TTGGGCTGTGTGTGGTGGCCCTGGTTCTGAGCTGGATGCACCTCGCCTCCGCTGGCAGCCGAGGGCTCAAGGGGA [150]
26268_T.europaea            ........................................................................... [150]
26948_T.europaea            ........................................................................... [150]
27078_T.europaea            ........................................................................... [150]
T23-15_T.europaea           ........................................................................... [150]
T23-16_T.europaea           ........................................................................... [150]
T23-17_T.europaea           ........................................................................... [150]
T23-18_T.europaea           ........................................................................... [150]

RCFO01000009_T.occidentalis AGAGGCAGAGGCGCAGTGAGTAGTGGCCGCGGGGCCACCCGGGCCTTGGGTGTGGAGGGGGCACTTGGGGAGTA [225]
26268_T.europaea            .......................-----............................................... [225]
26948_T.europaea            .......................-----............................................... [225]
27078_T.europaea            .......................-----............................................... [225]
T23-15_T.europaea           .......................-----............................................... [225]
T23-16_T.europaea           .......................-----............................................... [225]
T23-17_T.europaea           .......................-----............................................... [225]
T23-18_T.europaea           .......................-----............................................... [225]

RCFO01000009_T.occidentalis GAGGGCCTTCTGGGAGTTTCACTCTGTGAGG [256]
26268_T.europaea            ..............G.....G.......... [256]
26948_T.europaea            ..............G.....G.......... [256]
27078_T.europaea            ..............G.....G.......... [256]
T23-15_T.europaea           ..............G.....G.......... [256]
T23-16_T.europaea           ..............G.....G.......... [256]
T23-17_T.europaea           ..............G.....G.......... [256]
T23-18_T.europaea           ..............G.....G.......... [256]
```

Alignment S1,b. Alignment of nucleotide sequences of the *Rspo1* gene (the intermediate fragment II) of *T. occidentalis* and *T. europaea* specimens. The exon area is underlined in the upper sequence. The heterozygous sites, which were revealed in two of seven European moles, marked by blue.

```
RCFO01000009_T.occidentalis GCTAATTGCCTGGAGAGAGCATCCTGGGCGCTTTATAAAGCAGCCTGCCTGCTTGGCCTGTGCTTCCAAGAAG-A [ 75]
26268_T.europaea            .......C.................................................................C. [ 75]
26948_T.europaea            .......C.................................................................C. [ 75]
27078_T.europaea            .......C.................................................................C. [ 75]
T23-15_T.europaea           .......C.................................................................C. [ 75]
T23-16_T.europaea           .......C.................................................................C. [ 75]
T23-17_T.europaea           .......C.................................................................C. [ 75]
T23-18_T.europaea           .......C.................................................................C. [ 75]

RCFO01000009_T.occidentalis TGGTCGGTCTTCTCACCCCAGCCCGCGGGAGCCTGGGAAAGAGAGGTCCCTCCTTGCGCCGCCTTCTCACCCTAT [150]
26268_T.europaea            ...........A..............A................................................ [150]
26948_T.europaea            ...........A..............A................................................ [150]
27078_T.europaea            ...........A..............A................................................ [150]
T23-15_T.europaea           ...........A..............A................................................ [150]
T23-16_T.europaea           ...........A..............A................................................ [150]
T23-17_T.europaea           ...........A..............A................................................ [150]
T23-18_T.europaea           ...........A..............A................................................ [150]

RCFO01000009_T.occidentalis TCTGCCTTGCAGTCAGCACTGAAGGGAGCCAGGCCTGTGCCAAAGGCTGCGAACTCTGCTCCGAGGTCAACGGCT [225]
26268_T.europaea            ........................................................................... [225]
26948_T.europaea            ........................................................................... [225]
27078_T.europaea            ........................................................................... [225]
T23-15_T.europaea           ........................................................................... [225]
T23-16_T.europaea           ........................................................................... [225]
T23-17_T.europaea           ........................................................................... [225]
T23-18_T.europaea           ........................................................................... [225]

RCFO01000009_T.occidentalis GCCTCAAGTGCTCGCCCAAGCTGTTCATCCTGCTGGAGAGGAACGACATCCGCCAGGTGGGCGTCTGCTTGCCGT [300]
26268_T.europaea            ........................................................................... [300]
26948_T.europaea            ........................................................................... [300]
27078_T.europaea            ........................................................................... [300]
T23-15_T.europaea           ........................................................................... [300]
T23-16_T.europaea           ........................................................................... [300]
T23-17_T.europaea           ........................................................................... [300]
T23-18_T.europaea           ........................................................................... [300]
```

```
RCFO01000009_T.occidentalis CCTGCCCACCTGGATACTTCGATGCCCGCAGCCCCGACATGAACAAGTGCATCAGTGAGTGTGGCAGGGCTGGAG [375]
26268_T.europaea            ........................................................................... [375]
26948_T.europaea            ........................................................................... [375]
27078_T.europaea            ........................................................................... [375]
T23-15_T.europaea           ........................................................................... [375]
T23-16_T.europaea           ........................................................................... [375]
T23-17_T.europaea           ........................................................................... [375]
T23-18_T.europaea           ........................................................................... [375]

RCFO01000009_T.occidentalis AAGGGCATCAGAGGCATCGGGGTGCGTTGAGCTGCTGGTGGCTGATGAGAGGAACAGAGGGGACACCGAGGAGGG [450]
26268_T.europaea            ....................................T.............................T........ [450]
26948_T.europaea            ....................................T.............................T........ [450]
27078_T.europaea            ....................................T.............................T........ [450]
T23-15_T.europaea           ....................................T.............................T........ [450]
T23-16_T.europaea           ....................................T.............................T........ [450]
T23-17_T.europaea           ....................................T.............................T........ [450]
T23-18_T.europaea           ....................................T.............................T........ [450]

RCFO01000009_T.occidentalis GAGACGAGGCTCCTCGTTCCAGTTATCTGCTACCG [485]
26268_T.europaea            .....A............................. [485]
26948_T.europaea            .....A.........R................... [485]
27078_T.europaea            .....A............................. [485]
T23-15_T.europaea           .....A............................. [485]
T23-16_T.europaea           .....A.........R................... [485]
T23-17_T.europaea           .....A............................. [485]
T23-18_T.europaea           .....A............................. [485]
```

Alignment S1,c. Alignment of nucleotide sequences of the *Rspo1* gene (the final fragment III) of *T. occidentalis* and *T. europaea* specimens. The exon areas are underlined in the upper sequence.

```
RCFO01000009_T.occidentalis TTGGGAGTTGGCTGACCTTGGCAAGTCCATGTAGCCCTCAAGAGTGACCACCTTGCCCTCCCCCTGTCCTTGCCC [  75]
26268_T.europaea            ........................................................................... [  75]
26948_T.europaea            ........................................................................... [  75]
27078_T.europaea            ........................................................................... [  75]
T23-15_T.europaea           ........................................................................... [  75]
T23-16_T.europaea           ........................................................................... [  75]
T23-17_T.europaea           ........................................................................... [  75]
T23-18_T.europaea           ........................................................................... [  75]

RCFO01000009_T.occidentalis TTCCTTCCCCGCCCTTCCAAGACTTCGGGTCATTGCAACTCCAGGCCCCCACCCTATCTTCAATGTGGGTGGGGG [ 150]
26268_T.europaea            .........................T................................................. [ 150]
26948_T.europaea            .........................T................................................. [ 150]
27078_T.europaea            .........................T................................................. [ 150]
T23-15_T.europaea           .........................T................................................. [ 150]
T23-16_T.europaea           .........................T................................................. [ 150]
T23-17_T.europaea           .........................T................................................. [ 150]
T23-18_T.europaea           .........................T................................................. [ 150]

RCFO01000009_T.occidentalis TGGATGGCGCTGGGGATGGGGATGAAGGGAAGTGTCCATTCCAGGCTGAACCAGAGGCCCCCCATCTGGTCCTCT [ 225]
26268_T.europaea            ........................................................................... [ 225]
26948_T.europaea            ........................................................................... [ 225]
27078_T.europaea            ........................................................................... [ 225]
T23-15_T.europaea           ........................................................................... [ 225]
T23-16_T.europaea           ........................................................................... [ 225]
T23-17_T.europaea           ........................................................................... [ 225]
T23-18_T.europaea           ........................................................................... [ 225]

RCFO01000009_T.occidentalis CCTCAGAATGCAAGATTGAGCACTGCGAGGCCTGCTTCAGCCACAACTTCTGCACCAAGTGTAAGGAGAGCTTGT [ 300]
26268_T.europaea            ........................................................................... [ 300]
26948_T.europaea            ........................................................................... [ 300]
27078_T.europaea            ........................................................................... [ 300]
T23-15_T.europaea           ........................................................................... [ 300]
T23-16_T.europaea           ........................................................................... [ 300]
T23-17_T.europaea           ........................................................................... [ 300]
T23-18_T.europaea           ........................................................................... [ 300]
```

```
RCFO01000009_T.occidentalis ACCTGCACAAGGGCCGCTGCTACCCAGACTGCCCCGAGGGCTCTACAGCAGCCAACGGCACCATGGAGTGCAGCA [ 375]
26268_T.europaea            ........................................................................... [ 375]
26948_T.europaea            ........................................................................... [ 375]
27078_T.europaea            ........................................................................... [ 375]
T23-15_T.europaea           ........................................................................... [ 375]
T23-16_T.europaea           ........................................................................... [ 375]
T23-17_T.europaea           ........................................................................... [ 375]
T23-18_T.europaea           ........................................................................... [ 375]

RCFO01000009_T.occidentalis GTCCGGGTGAGGTGCCAGAGGCCTGGTAGGGGGCGGGGGTGGGGGTGGGGGGTGGGACGGCTGGCCTAGGAGAGC [ 450]
26268_T.europaea            ...........................-----------------------poly-G................... [ 450]
26948_T.europaea            ...........................-----------------------poly-G................... [ 450]
27078_T.europaea            ...........................-----------------------poly-G................... [ 450]
T23-15_T.europaea           ...........................-----------------------poly-G................... [ 450]
T23-16_T.europaea           ...........................-----------------------poly-G................... [ 450]
T23-17_T.europaea           ...........................-----------------------poly-G................... [ 450]
T23-18_T.europaea           ...........................-----------------------poly-G................... [ 450]

RCFO01000009_T.occidentalis AGAAATGTGCAGAGAAAGGTTACCCTACAAACACCCTCCGGGATTTCTCTTGGGACTTTTCTGCCCTTTGGACTA [ 525]
26268_T.europaea            ........................................................................... [ 525]
26948_T.europaea            ........................................................................... [ 525]
27078_T.europaea            ........................................................................... [ 525]
T23-15_T.europaea           ........................................................................... [ 525]
T23-16_T.europaea           ........................................................................... [ 525]
T23-17_T.europaea           ........................................................................... [ 525]
T23-18_T.europaea           ........................................................................... [ 525]

RCFO01000009_T.occidentalis AAGGTGCCTCCAGCCTAAGGCTTAGGCAAGAGCAGGTAGAAGAGGCTCCCCTGGGGTACCCTGGGTCATGGGAGG [ 600]
26268_T.europaea            ........................................................................... [ 600]
26948_T.europaea            ........................................................................... [ 600]
27078_T.europaea            ........................................................................... [ 600]
T23-15_T.europaea           ........................................................................... [ 600]
T23-16_T.europaea           ........................................................................... [ 600]
T23-17_T.europaea           ........................................................................... [ 600]
T23-18_T.europaea           ........................................................................... [ 600]
```

```
RCFO01000009_T.occidentalis AGACATGACCCCCTCCTGCTCTCTCTTTCCCTCTCCCCTCCCTGCCTGCCAGCACAATGTGAAATGAGCGAGTGG [ 675]
26268_T.europaea            ........................................................................... [ 675]
26948_T.europaea            ........................................................................... [ 675]
27078_T.europaea            ........................................................................... [ 675]
T23-15_T.europaea           ........................................................................... [ 675]
T23-16_T.europaea           ........................................................................... [ 675]
T23-17_T.europaea           ........................................................................... [ 675]
T23-18_T.europaea           ........................................................................... [ 675]

RCFO01000009_T.occidentalis TCCCCGTGGGGGCCGTGCTCCAAGAAGAAGAAGCTCTGTGGTTTCCGGAGGGGCTCTGAGGAGCGGACTCGGAGG [ 750]
26268_T.europaea            ........................................................................... [ 750]
26948_T.europaea            ........................................................................... [ 750]
27078_T.europaea            ........................................................................... [ 750]
T23-15_T.europaea           ........................................................................... [ 750]
T23-16_T.europaea           ........................................................................... [ 750]
T23-17_T.europaea           ........................................................................... [ 750]
T23-18_T.europaea           ........................................................................... [ 750]

RCFO01000009_T.occidentalis GTGCTCCATGCGCCTGGGGGGGACCATGCTACCTGCTCTGACATCAAGGAGACCCGAAGGTGCACGGTGATAAGG [ 825]
26268_T.europaea            ........................................................G.................. [ 825]
26948_T.europaea            ........................................................G.................. [ 825]
27078_T.europaea            ........................................................G.................. [ 825]
T23-15_T.europaea           ........................................................G.................. [ 825]
T23-16_T.europaea           ........................................................G.................. [ 825]
T23-17_T.europaea           ........................................................G.................. [ 825]
T23-18_T.europaea           ........................................................G.................. [ 825]

RCFO01000009_T.occidentalis ACTCCCTGTCCTGAAGGTAAGCTGCAGCCTCTGCCTCCCAGGGGCCCGGGGTCAGACACCGCCCCA-GACGTCTG [ 900]
26268_T.europaea            ..G.........................................-.....................G........ [ 900]
26948_T.europaea            ..G.........................................-.....................G........ [ 900]
27078_T.europaea            ..G.........................................-.....................G........ [ 900]
T23-15_T.europaea           ..G.........................................-.....................G........ [ 900]
T23-16_T.europaea           ..G.........................................-.....................G........ [ 900]
T23-17_T.europaea           ..G.........................................-.....................G........ [ 900]
T23-18_T.europaea           ..G.........................................-.....................G........ [ 900]
```

```
RCFO01000009_T.occidentalis AGGGCTCTGCTTCCTACCATTGCCTGGGAGAGGCCCTGAGGCCAAGGCCGCCTCAAGACACCCTGCTCTGCGAAC [ 975]
26268_T.europaea            ........................................................................... [ 975]
26948_T.europaea            ........................................................................... [ 975]
27078_T.europaea            ........................................................................... [ 975]
T23-15_T.europaea           ........................................................................... [ 975]
T23-16_T.europaea           ........................................................................... [ 975]
T23-17_T.europaea           ........................................................................... [ 975]
T23-18_T.europaea           ........................................................................... [ 975]

RCFO01000009_T.occidentalis CAGGACCCTGTCCTGGCAGAGCGGTCCAGGGCTCTGGGGGAGCTGGAAGCTTCGCGTCCCTATCCGTCCAGTTTC [1050]
26268_T.europaea            .................................................................A......... [1050]
26948_T.europaea            .................................................................A......... [1050]
27078_T.europaea            .................................................................A......... [1050]
T23-15_T.europaea           .................................................................A......... [1050]
T23-16_T.europaea           .................................................................A......... [1050]
T23-17_T.europaea           .................................................................A......... [1050]
T23-18_T.europaea           .................................................................A......... [1050]

RCFO01000009_T.occidentalis CTCTTTTCTTCTCAGTCAGTGATATTGGTTCTGGAGTTCAGAGGGGGGTCCAGGAATGACACAAAGTCTCACGGA [1125]
26268_T.europaea            ...........................................C............................A.. [1125]
26948_T.europaea            ...........................................C............................A.. [1125]
27078_T.europaea            ...........................................C............................A.. [1125]
T23-15_T.europaea           ...........................................C............................A.. [1125]
T23-16_T.europaea           ...........................................C............................A.. [1125]
T23-17_T.europaea           ...........................................C............................A.. [1125]
T23-18_T.europaea           ...........................................C............................A.. [1125]

RCFO01000009_T.occidentalis GGTGGAGTGATTTGACCAAGGTCACGCAGTCAGCCACTGGAAATGATGTGGCAGCTGGCCCCAGAGGCAGTTGTG [1200]
26268_T.europaea            ..................................T........................................ [1200]
26948_T.europaea            ..................................T........................................ [1200]
27078_T.europaea            ..................................T........................................ [1200]
T23-15_T.europaea           ..................................T........................................ [1200]
T23-16_T.europaea           ..................................T........................................ [1200]
T23-17_T.europaea           ..................................T........................................ [1200]
T23-18_T.europaea           ..................................T........................................ [1200]
```

```
RCFO01000009_T.occidentalis TGGGGCAGAGTCGT-GGGGGGTCTTCCTGCCTGTTTTGAGACTTGCAATGAACATCTAGGGAGTGCTGTTGGCCC [1275]
26268_T.europaea            ..............G............................................................ [1275]
26948_T.europaea            ..............G............................................................ [1275]
27078_T.europaea            ..............G............................................................ [1275]
T23-15_T.europaea           ..............G............................................................ [1275]
T23-16_T.europaea           ..............G............................................................ [1275]
T23-17_T.europaea           ..............G............................................................ [1275]
T23-18_T.europaea           ..............G............................................................ [1275]

RCFO01000009_T.occidentalis ACTGGGACCCAGAGGGAGGAAGACATGAGGAAGAGGAAGGTACAAAGGCTCTTGGGACCTCCTCTCAGGACCCAA [1350]
26268_T.europaea            ........................................................................... [1350]
26948_T.europaea            ........................................................................... [1350]
27078_T.europaea            ........................................................................... [1350]
T23-15_T.europaea           ........................................................................... [1350]
T23-16_T.europaea           ........................................................................... [1350]
T23-17_T.europaea           ........................................................................... [1350]
T23-18_T.europaea           ........................................................................... [1350]

RCFO01000009_T.occidentalis GTTGTAGGGTCCTCTGAATTGGTTTCTGGTCAGAGGGGGCCTCCAGGTGGCATCTCCAGGCATCCTCCTGCCTTC [1425]
26268_T.europaea            .......................................................................G... [1425]
26948_T.europaea            .......................................................................G... [1425]
27078_T.europaea            .......................................................................G... [1425]
T23-15_T.europaea           .......................................................................G... [1425]
T23-16_T.europaea           .......................................................................G... [1425]
T23-17_T.europaea           .......................................................................G... [1425]
T23-18_T.europaea           .......................................................................G... [1425]

RCFO01000009_T.occidentalis CCCCCAGGCCAGCTTCCTCACCACTCCCCGCTCAGGCCCTTGGCCATGCCCAGGACCTGATTGCTTGTGGTGGGA [1500]
26268_T.europaea            ........................................................................... [1500]
26948_T.europaea            ........................................................................... [1500]
27078_T.europaea            ........................................................................... [1500]
T23-15_T.europaea           ........................................................................... [1500]
T23-16_T.europaea           ........................................................................... [1500]
T23-17_T.europaea           ........................................................................... [1500]
T23-18_T.europaea           ........................................................................... [1500]
```

```
RCFO01000009_T.occidentalis CAGTCTTTGATGACCCATTCTACTGTTGGCGTTGCAGGGCAGAAAAGGAGGAAAGGCGGCCAGGGCCGGCGGGAG [1575]
26268_T.europaea            ........................................................................... [1575]
26948_T.europaea            ........................................................................... [1575]
27078_T.europaea            ........................................................................... [1575]
T23-15_T.europaea           ........................................................................... [1575]
T23-16_T.europaea           ........................................................................... [1575]
T23-17_T.europaea           ........................................................................... [1575]
T23-18_T.europaea           ........................................................................... [1575]

RCFO01000009_T.occidentalis AATGCCAACAGGAGCCTGAACAGGAAGGAGAACAAGGAGGCGGGCACTGCAAGGAGACGCAAGGGCCAGCAGCAG [1650]
26268_T.europaea            ........................................................................... [1650]
26948_T.europaea            ........................................................................... [1650]
27078_T.europaea            ........................................................................... [1650]
T23-15_T.europaea           ........................................................................... [1650]
T23-16_T.europaea           ........................................................................... [1650]
T23-17_T.europaea           ........................................................................... [1650]
T23-18_T.europaea           ........................................................................... [1650]

RCFO01000009_T.occidentalis CAGCAGCATCAAGGGACAGTGGGGCCAGTCACATCTCCAGGGCCCACTTAGGGACACTGTTCAGCCTCCAGGCCC [1725]
26268_T.europaea            ........................................................................... [1725]
26948_T.europaea            ........................................................................... [1725]
27078_T.europaea            ........................................................................... [1725]
T23-15_T.europaea           ........................................................................... [1725]
T23-16_T.europaea           ........................................................................... [1725]
T23-17_T.europaea           ........................................................................... [1725]
T23-18_T.europaea           ........................................................................... [1725]

RCFO01000009_T.occidentalis ATGCAGAAAAAGTCCAGCGCAGCTCTGTGTGATGAAAGCCTTACTGAAATGGAGTGGCAAGGGCAATGCACACAC [1800]
26268_T.europaea            ........................................................................... [1800]
26948_T.europaea            ........................................................................... [1800]
27078_T.europaea            ........................................................................... [1800]
T23-15_T.europaea           ........................................................................... [1800]
T23-16_T.europaea           ........................................................................... [1800]
T23-17_T.europaea           ........................................................................... [1800]
T23-18_T.europaea           ........................................................................... [1800]
```

```
RCFO01000009_T.occidentalis AGTCTCCTACATACATGCACGCGCACAGACCCCATGTCCACATATGCAATCAACAAACATGCACACAAGTGTGCA [1875]
26268_T.europaea            ...................T....................................................... [1875]
26948_T.europaea            ...................T....................................................... [1875]
27078_T.europaea            ...................T....................................................... [1875]
T23-15_T.europaea           ...................T....................................................... [1875]
T23-16_T.europaea           ...................T....................................................... [1875]
T23-17_T.europaea           ...................T....................................................... [1875]
T23-18_T.europaea           ...................T....................................................... [1875]

RCFO01000009_T.occidentalis CGTGCACACTCAAGGCCACTGGAATAC [1902]
26268_T.europaea            ........................... [1902]
26948_T.europaea            ........................... [1902]
27078_T.europaea            ........................... [1902]
T23-15_T.europaea           ........................... [1902]
T23-16_T.europaea           ........................... [1902]
T23-17_T.europaea           ........................... [1902]
T23-18_T.europaea           ........................... [1902]
```

Alignment S1,d. Alignment of nucleotide sequences corresponding to the *Rspo1* gene exons of several species of the Talpidae family (*Condylura cristata*, *Talpa occidentalis*, and *T. europaea*) and the Soricidae family (*Suncus etruscus*, *Sorex araneus*, *Sorex fumeus*, and *Sorex palustris*). Sequence of the predicted protein-coding part of the *Suncus etruscus* *Rspo1* gene is partially represented.

```
XM_004678811_C.cristata         ATGCGGCTTGGGCTGTGTGTGGTGGCCCTGGTTCTGAGCTGGATGCACCTCGCCTCCGCTGGCAGCCGAGGGCTC [ 75]
RCFO01000009_T.occidentalis     ........................................................................... [ 75]
26268_T.europaea                ........................................................................... [ 75]
26948_T.europaea                ........................................................................... [ 75]
27078_T.europaea                ........................................................................... [ 75]
T23-15_T.europaea               ........................................................................... [ 75]
T23-16_T.europaea               ........................................................................... [ 75]
T23-17_T.europaea               ........................................................................... [ 75]
T23-18_T.europaea               ........................................................................... [ 75]
XM_004614239_S.araneus          ......................CC.......................T........G..G........G...A.G [ 75]
JAOXYA010000032_S.palustris     ............T.........CC.......................T........G..A........G.....G [ 75]
XM_056123613_S.fumeus           ............T.........CC.......................T........G..A........G.....G [ 75]
XM_049775528_S.etruscus(part.)  ......................CC.......................T...T......AG..T.....G...A.. [ 75]

XM_004678811_C.cristata         AAGGGGAAGAGGCAGAGGCGCATCAGCGCTGAGGGGAGTCAGGCCTGCGCCAAGGGCTGTGAACTCTGCTCCGAG [150]
RCFO01000009_T.occidentalis     ...........................A....A.....C........T.....A.....C............... [150]
26268_T.europaea                ...........................A....A.....C........T.....A.....C............... [150]
26948_T.europaea                ...........................A....A.....C........T.....A.....C............... [150]
27078_T.europaea                ...........................A....A.....C........T.....A.....C............... [150]
T23-15_T.europaea               ...........................A....A.....C........T.....A.....C............... [150]
T23-16_T.europaea               ...........................A....A.....C........T.....A.....C............... [150]
T23-17_T.europaea               ...........................A....A.....C........T.....A.....C............... [150]
T23-18_T.europaea               ...........................A....A.....C........T.....A.....C............... [150]
XM_004614239_S.araneus          ....................G.....T..C........C.......................G...........A [150]
JAOXYA010000032_S.palustris     ....................G........C........C.......................G...........A [150]
XM_056123613_S.fumeus           ....................G........C........C.......................G...........A [150]
XM_049775528_S.etruscus(part.)  ....................G.....T..C........C..............A........G...........A [150]
```

```
XM_004678811_C.cristata          GTCAACGGCTGCCTCAAGTGCTCGCCCAAGCTGTTCATCCTGCTGGAGAGGAACGACATCCGCCAGGTGGGCGTC [225]
RCFO01000009_T.occidentalis      ........................................................................... [225]
26268_T.europaea                 ........................................................................... [225]
26948_T.europaea                 ........................................................................... [225]
27078_T.europaea                 ........................................................................... [225]
T23-15_T.europaea                ........................................................................... [225]
T23-16_T.europaea                ........................................................................... [225]
T23-17_T.europaea                ........................................................................... [225]
T23-18_T.europaea                ........................................................................... [225]
XM_004614239_S.araneus           ...........T....................C...............C................A......... [225]
JAOXYA010000032_S.palustris      ...........T....................C..T....................................A.. [225]
XM_056123613_S.fumeus            ...........T....................C.......................................A.. [225]
XM_049775528_S.etruscus(part.)   ...........T..G........A........C.......................................... [225]

XM_004678811_C.cristata          TGCTTGCCGTCCTGCCCACCTGGATACTTCGATGCCCGCAACCCCGACATGAACAAGTGCATCAAATGCAAGATC [300]
RCFO01000009_T.occidentalis      ........................................G.................................T [300]
26268_T.europaea                 ........................................G.................................T [300]
26948_T.europaea                 ........................................G.................................T [300]
27078_T.europaea                 ........................................G.................................T [300]
T23-15_T.europaea                ........................................G.................................T [300]
T23-16_T.europaea                ........................................G.................................T [300]
T23-17_T.europaea                ........................................G.................................T [300]
T23-18_T.europaea                ........................................G.................................T [300]
XM_004614239_S.araneus           ...C....C........G..G..T.........................................G....G.... [300]
JAOXYA010000032_S.palustris      ...C.............G..A..T.................T.......................G....G.... [300]
XM_056123613_S.fumeus            ...C.............G..G..T.................T.......................G....G.... [300]
XM_049775528_S.etruscus(part.)   ...C....C........T..C..T.........................................G........T [300]

XM_004678811_C.cristata          GAGCACTGCGAGGCCTGCTTCAGCCACAACTTCTGCACCAAGTGTAAGGAGAACTTGTACCTGCACAAGGGCCGC [375]
RCFO01000009_T.occidentalis      ....................................................G...................... [375]
26268_T.europaea                 ....................................................G...................... [375]
26948_T.europaea                 ....................................................G...................... [375]
27078_T.europaea                 ....................................................G...................... [375]
T23-15_T.europaea                ....................................................G...................... [375]
T23-16_T.europaea                ....................................................G...................... [375]
T23-17_T.europaea                ....................................................G...................... [375]
T23-18_T.europaea                ....................................................G...................... [375]
XM_004614239_S.araneus           ..............A.............................C.GA....G.C...T................ [375]
JAOXYA010000032_S.palustris      ..............A.............................C.G.....G.C...T................ [375]
XM_056123613_S.fumeus            ..............A.............................C.GA......C...T................ [375]
XM_049775528_S.etruscus(part.)   ..A...........A.......................G.....C.G...A.G.C...TT...........T... [375]
```

```
XM_004678811_C.cristata         TGCTACCCGGCCTGCCCCGAGGGCTCTGCAGCGCCCAACAGCACCATGGAGTGCAGCAGTCCGGCACAATGTGAA [450]
RCFO01000009_T.occidentalis     ........A.A................A....AG.....G................................... [450]
26268_T.europaea                ........A.A................A....AG.....G................................... [450]
26948_T.europaea                ........A.A................A....AG.....G................................... [450]
27078_T.europaea                ........A.A................A....AG.....G................................... [450]
T23-15_T.europaea               ........A.A................A....AG.....G................................... [450]
T23-16_T.europaea               ........A.A................A....AG.....G................................... [450]
T23-17_T.europaea               ........A.A................A....AG.....G................................... [450]
T23-18_T.europaea               ........A.A................A....AG.....G................................... [450]
XM_004614239_S.araneus          .......G..................CA.CC..G.....G..............G..CAC..C.G.......... [450]
JAOXYA010000032_S.palustris     .......G..................CA.C..TGT....G..............G..CAC..T.G.......... [450]
XM_056123613_S.fumeus           .......G..................CA.C..TGT....G..............G..CAC..T.G.......... [450]
XM_049775528_S.etruscus(part.)  .......TT........A........CA.T..TG.....G.............TG...AC..T............ [450]

XM_004678811_C.cristata         ATGAGCGAGTGGTCCCCATGGGGTCCATGCTCCAAGAAGAAGAAGCTCTGTGGTTTCCGGAAGGGCTCTGAGGAG [525]
RCFO01000009_T.occidentalis     .................G.....G..G..................................G............. [525]
26268_T.europaea                .................G.....G..G..................................G............. [525]
26948_T.europaea                .................G.....G..G..................................G............. [525]
27078_T.europaea                .................G.....G..G..................................G............. [525]
T23-15_T.europaea               .................G.....G..G..................................G............. [525]
T23-16_T.europaea               .................G.....G..G..................................G............. [525]
T23-17_T.europaea               .................G.....G..G..................................G............. [525]
T23-18_T.europaea               .................G.....G..G..................................G............. [525]
XM_004614239_S.araneus          C.............G..G.....G.TG..........G...............C.......G......A...... [525]
JAOXYA010000032_S.palustris     C.A..T........G..G.....G..G..........G...............C.......G......A...... [525]
XM_056123613_S.fumeus           C.A..T........G..G.....G..G..........G...............C.......G......A...... [525]
XM_049775528_S.etruscus(part.)  C....T..A.....A..C.....G........T....GA......................G....A.C...... [525]

XM_004678811_C.cristata         CGGACGCGGAGGGTGCTCCATGCCTCTGGGGGGACCTCGCCCCCTGCACCGACATCAAGGAGACCCGGAGGTGC [600]
RCFO01000009_T.occidentalis     .....T.................GC............AT..TA.....T.T.................A...... [600]
26268_T.europaea                .....T.................GC............AT..TA.....T.T........................ [600]
26948_T.europaea                .....T.................GC............AT..TA.....T.T........................ [600]
27078_T.europaea                .....T.................GC............AT..TA.....T.T........................ [600]
T23-15_T.europaea               .....T.................GC............AT..TA.....T.T........................ [600]
T23-16_T.europaea               .....T.................GC............AT..TA.....T.T........................ [600]
T23-17_T.europaea               .....T.................GC............AT..TA.....T.T........................ [600]
T23-18_T.europaea               .....T.................GC............AT..TA.....T.T........................ [600]
XM_004614239_S.araneus          .........C.......G..C...C.C..........A.A..TT....T.G.G..C.........T......... [600]
JAOXYA010000032_S.palustris     .....A...C.......G..A...C.......A....ATA..TT....T.A.G..C.........T......... [600]
XM_056123613_S.fumeus           .....A...C.......G..A...C.......A....A.A..TT....T.A.G..C.........T......... [600]
XM_049775528_S.etruscus(part.)  A..T..A....A.....G.....GC....A.......A.A..AT....T.G....C................... [600]
```

```
XM_004678811_C.cristata          ACAGTGCGGAGGACGCCCTGTTCTGAAGGGCAGAAGAGGAGGAAAGGCGGCCAGGGCCGGCGGGAGAAGGCCAGC [675]
RCFO01000009_T.occidentalis      ..G...ATA.....T......C.............A................................T....A. [675]
26268_T.europaea                 ..G...ATA............C.............A................................T....A. [675]
26948_T.europaea                 ..G...ATA............C.............A................................T....A. [675]
27078_T.europaea                 ..G...ATA............C.............A................................T....A. [675]
T23-15_T.europaea                ..G...ATA............C.............A................................T....A. [675]
T23-16_T.europaea                ..G...ATA............C.............A................................T....A. [675]
T23-17_T.europaea                ..G...ATA............C.............A................................T....A. [675]
T23-18_T.europaea                ..G...ATA............C.............A................................T....A. [675]
XM_004614239_S.araneus           .................G...C...................A..G..G..........A......C..C....AG [675]
JAOXYA010000032_S.palustris      ..G..A........A..A...C......................G..G..........A......C..C....AG [675]
XM_056123613_S.fumeus            ..G..A........A..G...C......................G..G..........A......C..C....AG [675]
XM_049775528_S.etruscus(part.)   .....T.....A..A..A...C......................G..G.....A....A......CGGT....AG [675]

XM_004678811_C.cristata          AGGAACCTGAGCAGGAAGGAGAGCAATGAGGCGGGCGCT---GCCAGGAGACGCAAGGGCCAGCAGCCGCCACCG [750]
RCFO01000009_T.occidentalis      ....G.....A...........A...G.........A..---..A......................A..AG.A. [750]
26268_T.europaea                 ....G.....A...........A...G.........A..---..A......................A..AG.A. [750]
26948_T.europaea                 ....G.....A...........A...G.........A..---..A......................A..AG.A. [750]
27078_T.europaea                 ....G.....A...........A...G.........A..---..A......................A..AG.A. [750]
T23-15_T.europaea                ....G.....A...........A...G.........A..---..A......................A..AG.A. [750]
T23-16_T.europaea                ....G.....A...........A...G.........A..---..A......................A..AG.A. [750]
T23-17_T.europaea                ....G.....A...........A...G.........A..---..A......................A..AG.A. [750]
T23-18_T.europaea                ....G.....A...........A...G.........A..---..A......................A..AG.A. [750]
XM_004614239_S.araneus           .....GTCA...C.............G....T....ATCGGCT..C........C......C.....A..AG... [750]
JAOXYA010000032_S.palustris      .....GTCA...C.............G.........ATCGGCT..C...............C........AG... [750]
XM_056123613_S.fumeus            .....GTCA...C.............G....A....ATCGGCT..C.............T.C......A.AG... [750]
XM_049775528_S.etruscus(part.)   .....ATCA.A.C.A...........G....T....A.CAGCT..CA...........CT------------.T. [750]

XM_004678811_C.cristata          CAGCAAGGGACAGTGGGGCCCGTCACGTCTGCGGGGCCCACCTAG [795]
RCFO01000009_T.occidentalis      ..T.................A.....A...C.A........T... [795]
26268_T.europaea                 ..T.................A.....A...C.A........T... [795]
26948_T.europaea                 ..T.................A.....A...C.A........T... [795]
27078_T.europaea                 ..T.................A.....A...C.A........T... [795]
T23-15_T.europaea                ..T.................A.....A...C.A........T... [795]
T23-16_T.europaea                ..T.................A.....A...C.A........T... [795]
T23-17_T.europaea                ..T.................A.....A...C.A........T... [795]
T23-18_T.europaea                ..T.................A.....A...C.A........T... [795]
XM_004614239_S.araneus           G.C.G.......A.....------..A---.T............. [795]
JAOXYA010000032_S.palustris      G.C.G.............------..A---.T............. [795]
XM_056123613_S.fumeus            G.C.G.............------.....---.T............. [795]
XM_049775528_S.etruscus(part.)   ..C.G...A---------..A------------------------ [795]
```

Alignment S2. Alignment of nucleotide sequences of the *Eif2s3x* and *Eif2s3y* genes of *T. occidentalis* and *T. europaea* specimens. The exon areas are underlined in the upper sequence.

```
RCFO01000015_T.occidentalis ACAGTTGTCAAAGCTATTTCCGGAGTTCACACTGTCCGGTTCAAAAATGAACTAGAAAGAAACATTACAATCAAA [ 75]
26268_T.europaea_(Eif2s3x)  ........................................................................... [ 75]
26906_T.europaea_(Eif2s3x)  ........................................................................... [ 75]
26948_T.europaea_(Eif2s3x)  ........................................................................... [ 75]
27078_T.europaea_(Eif2s3x)  ........................................................................... [ 75]
T23-15_T.europaea_(Eif2s3x) ........................................................................... [ 75]
T23-16_T.europaea_(Eif2s3x) ........................................................................... [ 75]
T23-17_T.europaea_(Eif2s3x) ........................................................................... [ 75]
T23-18_T.europaea_(Eif2s3x) ........................................................................... [ 75]
RCFO01000018_T.occidentalis ........A...........T........T......A................G........T.....T...... [ 75]
T23-16_T.europaea_(Eif2s3y) ........A...........T........T......A................G........T.....T...... [ 75]
T23-18_T.europaea_(Eif2s3y) ........A...........T........T......A................G........T.....T...... [ 75]

RCFO01000015_T.occidentalis CTTGGATATGCCAACGCTAAGGTAAGCTATATACTGTGGAACTGGAAACAAACTTTAACAGTATGCAAATAACAA [150]
26268_T.europaea_(Eif2s3x)  ........................................................................... [150]
26906_T.europaea_(Eif2s3x)  ........................................................................... [150]
26948_T.europaea_(Eif2s3x)  ........................................................................... [150]
27078_T.europaea_(Eif2s3x)  ........................................................................... [150]
T23-15_T.europaea_(Eif2s3x) ........................................................................... [150]
T23-16_T.europaea_(Eif2s3x) ........................................................................... [150]
T23-17_T.europaea_(Eif2s3x) ........................................................................... [150]
T23-18_T.europaea_(Eif2s3x) ........................................................................... [150]
RCFO01000018_T.occidentalis ..............T..C......CATAGGC.....G....G.A..T.TCTCA..ACTTT..T---.....TGT. [150]
T23-16_T.europaea_(Eif2s3y) ..............T..C......CATAGGC.G...G....G.A..T.TC.CG..ACTTT..T---.....TGT. [150]
T23-18_T.europaea_(Eif2s3y) ..............T..C......CATAGGC.G...G....G.A..T.TC.CG..ACTTT..T---.....TGT. [150]

RCFO01000015_T.occidentalis ATCCAAATCTTTTGTTCTT-CATTGAAACGTTTAGATTTACAAACTTGATGACCCAAGTTGTCCTCGTCCAGAAT [225]
26268_T.europaea_(Eif2s3x)  ...................T...............................................G....... [225]
26906_T.europaea_(Eif2s3x)  ...................T...............................................G....... [225]
26948_T.europaea_(Eif2s3x)  ...................T...............................................G....... [225]
27078_T.europaea_(Eif2s3x)  ...................T...............................................G....... [225]
T23-15_T.europaea_(Eif2s3x) ...................T...............................................G....... [225]
T23-16_T.europaea_(Eif2s3x) ...................T...............................................G....... [225]
T23-17_T.europaea_(Eif2s3x) ...................T...............................................G....... [225]
T23-18_T.europaea_(Eif2s3x) ...................T...............................................G....... [225]
RCFO01000018_T.occidentalis .ATT.C.AA...A..CT..T..C......---........T...........TG........G....G....... [225]
T23-16_T.europaea_(Eif2s3y) .ATT.C.AA...A..CT..T..C......---........T...........TG........G....G....... [225]
T23-18_T.europaea_(Eif2s3y) .ATT.C.AA...A..CT..T..C......---........T...........TG........G....G....... [225]
```

```
RCFO01000015_T.occidentalis GTTACAGATCTTGTGGAAGTAG [247]
26268_T.europaea_(Eif2s3x)  ...................... [247]
26906_T.europaea_(Eif2s3x)  ...................... [247]
26948_T.europaea_(Eif2s3x)  ...................... [247]
27078_T.europaea_(Eif2s3x)  ...................... [247]
T23-15_T.europaea_(Eif2s3x) ...................... [247]
T23-16_T.europaea_(Eif2s3x) ...................... [247]
T23-17_T.europaea_(Eif2s3x) ...................... [247]
T23-18_T.europaea_(Eif2s3x) ...................... [247]
RCFO01000018_T.occidentalis .C..TC....C........... [247]
T23-16_T.europaea_(Eif2s3y) .C..TC....C.           [247]
T23-18_T.europaea_(Eif2s3y) .C..TC....C.           [247]
```

Alignment S3,a. Alignment of nucleotide sequences of the *Sry* gene of *T. occidentalis* and *T. europaea* specimens. The exon areas are underlined in the upper sequence. The nucleotide sites, in which double peaks were observed in chromatograms, are marked by blue. The sites, demonstrating differences between the analyzed European mole males, are marked by a red asterisk. The area, showing poor homology to LINE-L1 retroposon, is marked by yellow.

```
RCFO01000018_T.occidentalis TCTGCCCCTGGAGAAACCTGCCGCCTGCTGCTGCAACGGGAGCTCTGGGGAACCCTTCCCAGAGTGAACCCAAGG [  75]
T23-16_T.europaea           .....................................A................T.................... [  75]
T23-18_T.europaea           .....................................A................T.................... [  75]

RCFO01000018_T.occidentalis ATCCTGCGCTCCAAACCCACTGACTCCAAAACCACAACAAAAGCACTGGAGAAGTAATTCAAAAGCCTGATATAT [ 150]
T23-16_T.europaea           .C....Y.................................................................... [ 150]
T23-18_T.europaea           .C....Y.................................................................... [ 150]

RCFO01000018_T.occidentalis GCCAGCCACTATTCTCATGATCTAGACATTCAAAACAAAGGAAGCCTCTGTTTTTCTTTTACATTAGATTCCATT [ 225]
T23-16_T.europaea           ..................................................Y........................ [ 225]
T23-18_T.europaea           ..................................................Y........................ [ 225]

RCFO01000018_T.occidentalis GATTGCTGCCTACGTGGCTCCTCCTGCCTTCATATGAAGTTGCAGATGTCCCTGGGGTAGTATTTGCCCTTTGCC [ 300]
T23-16_T.europaea           ..............................................................W............ [ 300]
T23-18_T.europaea           ..............................................................W............ [ 300]

RCFO01000018_T.occidentalis CCCAGTATGACTAAATACGCCAAACATAGAATCCACCATTTTTCACCCTTTTAAAGTATACAATTCACTAGCTTT [ 375]
T23-16_T.europaea           ...............................................A........................... [ 375]
T23-18_T.europaea           ...............................................A........................... [ 375]

RCFO01000018_T.occidentalis GAGGACAATTGCCAAAATATACAACAATCAGCTGCTTCTACTTCCGGAATATTTGGAATGATGGAATAAACATCA [ 450]
T23-16_T.europaea           .......R.....................................R............................. [ 450]
T23-18_T.europaea           .......R.....................................R............................. [ 450]

                             *
RCFO01000018_T.occidentalis CTGCAGAAGGAAATCCTGAGCAGTATTCCCTCCCCTGTTACCAGTCCCAGAGAATCACTAATCTGGATCCTGTGT [ 525]
T23-16_T.europaea           .A......................................................................... [ 525]
T23-18_T.europaea           .W......................................................................... [ 525]

RCFO01000018_T.occidentalis CCATGAATCTGCCTCTTGTGAGTATTTCATACAAATGGAATCTTTTCAGTATGTACCCTCTCGTTGTGTATCTAC [ 600]
T23-16_T.europaea           ..........................................-......C......................... [ 600]
T23-18_T.europaea           ..........................................-......C......................... [ 600]

RCFO01000018_T.occidentalis TTTTCTTTCGCACAGGATAATGTTTTTCCATGTTGTAGTACATAACACTTTTATTTTTATTTTTTTTTTTTGCCT [ 675]
T23-16_T.europaea           -........R...........................................------------poly-T.... [ 675]
T23-18_T.europaea           -........R...........................................------------poly-T.... [ 675]
```

```
RCFO01000018_T.occidentalis TTTTAATTCCGGTTTTGAAATAGTATATTCTTTTCTCATATTTCCCATGATGCCCAGTTTTACAAAGTCTGTTTT [ 750]
T23-16_T.europaea           ...................................................A....................... [ 750]
T23-18_T.europaea           ...................................................A....................... [ 750]

RCFO01000018_T.occidentalis TCAGTAAGAGTATAGTTTAAACATTTGATTCTGTGTTATAATATTTTAAATTGTGTAAAATACCATACTCACACC [ 825]
T23-16_T.europaea           ........................................................................... [ 825]
T23-18_T.europaea           ........................................................................... [ 825]

RCFO01000018_T.occidentalis TAATCTGTATCTTGAATACTAATTTTATTAGATGGCTAAGATACAAGTTGTGAGATTTACAGTAGTGTTTTGAGT [ 900]
T23-16_T.europaea           .................................................A...........C............. [ 900]
T23-18_T.europaea           .................................................A...........C............. [ 900]

RCFO01000018_T.occidentalis TTGTGATTTCATGCATAATAACATCTATTGCAAAAATGACAGTTGATTACATGGATTGTTTGCATGTAATGCGAA [ 975]
T23-16_T.europaea           ..........................................................G................ [ 975]
T23-18_T.europaea           ..........................................................G................ [ 975]

RCFO01000018_T.occidentalis CGTTTTTTAAATGTTATTTGCCTAGAGATTTTACATCATTCTTTGTGTTTAACCTTGAAGTGAATTTATTTTGTA [1050]
T23-16_T.europaea           ...C....................................................................... [1050]
T23-18_T.europaea           ...C....................................................................... [1050]

RCFO01000018_T.occidentalis CTTTTTAAAAAAAGATTTATTTATTTGCTCATTTATTTTATACAAGCACTGGGTACAGTCAGAAGCGCGGGAGGG [1125]
T23-16_T.europaea           ...............................C.....................C..................... [1125]
T23-18_T.europaea           ...............................C.....................C..................... [1125]

RCFO01000018_T.occidentalis TGAGAGAATCCACCAGAGCCAGAGCGGGGAGCAGA--AGG----------------------------------- [1200]
T23-16_T.europaea           ......G.CT.........................GC...CAGAAGGACCGAGGTACACTGCTGCGGGGAGCAGC [1200]
T23-18_T.europaea           ......G.CT.........................GC...CAGAAGGACCGAGGTACACTGCTGCGGGGAGCAGC [1200]

RCFO01000018_T.occidentalis ----GGCAGGAGAGGTCTGCGCGCCATGTGGGACGGTCGCCAGCGGCCAGGGTTTGAACCGGCGCAGCAGAGGCT [1275]
T23-16_T.europaea           GGGC..............---------................................A............... [1275]
T23-18_T.europaea           GGGC..............---------................................A............... [1275]

RCFO01000018_T.occidentalis GCGGGCAGCCTC-GCAACCCAGCGCTCAACCGCTGTGCCACCAGGGGAGGCCCTATTTTTACTTGTAATGGACTG [1350]
T23-16_T.europaea           ..T.........T...................................................T.......... [1350]
T23-18_T.europaea           ..T.........T...................................................T.......... [1350]

RCFO01000018_T.occidentalis TCGTTGTCAGTCACAATTTTGCAAACGTGTCTGGGAGAGGGGTTCTCCTATTTTATTTCATCCAAGTATTTTGTG [1425]
T23-16_T.europaea           .S......................................................................... [1425]
T23-18_T.europaea           .S......................................................................... [1425]
```

```
RCFO01000018_T.occidentalis ACAGTTTCCAAGTGTTTCCTCTTGCATGTATTTGGAGAAGGTATTCACCTACTTCACTAGGTAAAGCATGACCTT [1500]
T23-16_T.europaea           ................C........................C................................. [1500]
T23-18_T.europaea           ................C........................C................................. [1500]

RCFO01000018_T.occidentalis TGCATTTTGCCCTAAGTTTGAGCTAAAATATAAAAGCTGATAAGTTGTTCGCTCT-AACTTAGAAATTTAATTTA [1575]
T23-16_T.europaea           ..............C...................................A....T................... [1575]
T23-18_T.europaea           ..............C...................................A....T................... [1575]

RCFO01000018_T.occidentalis GATCTGTACAGCTATTTTTTAAATATTTGTTATGTGAAAGCACACGGAGGAGAGGCGCTTAAAGGTAAGACCTGT [1650]
T23-16_T.europaea           ............................................................G.............. [1650]
T23-18_T.europaea           ............................................................G.............. [1650]

RCFO01000018_T.occidentalis CTCCAGCCTCCTAAAGAAAATTCAGTCCCTTTGAAGTGTTACCTCAGCTGTCTTCTAACATCTAATTTGAATTGT [1725]
T23-16_T.europaea           .........................................T.....G........................... [1725]
T23-18_T.europaea           .........................................T.....G........................... [1725]

RCFO01000018_T.occidentalis TCCAGAGGCATTGAAATAACTGTAAAGTGTACATTAATATTTTCAGCATCGTTAGCATAGATAATAATATTAGTA [1800]
T23-16_T.europaea           ................................G....C....-.......T..................C..... [1800]
T23-18_T.europaea           ................................G....C....-.......T..................C..... [1800]

RCFO01000018_T.occidentalis GTAACGTTTTCAATTATTTTTAAAGGAGGAGTGGAGAAACAGTAGAGTGAGGATCACGGTGAGTTCAACCGTGCC [1875]
T23-16_T.europaea           .....T.............................................C......................G [1875]
T23-18_T.europaea           .....T.............................................C......................G [1875]

RCFO01000018_T.occidentalis TGTCTGCGTGCGTATCTGTGTAGTCGCGCGTATGAAAGGCCGATAAAAGGTGTGTAAGATTTGTGTTTCAGGTAA [1950]
T23-16_T.europaea           .............................C........................................C.... [1950]
T23-18_T.europaea           .............................C........................................C.... [1950]

RCFO01000018_T.occidentalis CATTCACGTGGATTCATGATGATAAGCAACCCGTTAGTATCTCTTTACTATACCTTCGCCTTTGATTTAAAGGGG [2025]
T23-16_T.europaea           ................................A.......................................... [2025]
T23-18_T.europaea           ................................A.......................................... [2025]

RCFO01000018_T.occidentalis TAATGATTTTATTAAAGTCCGTGTTCATAAAGAGAGGGTAGAGCCTTCAGTTATGCGGATTAGAGTCCAACACAA [2100]
T23-16_T.europaea           ..................................G...............................G-G...... [2100]
T23-18_T.europaea           ..................................G...............................G-G...... [2100]

RCFO01000018_T.occidentalis ACACAGCTGGGTGTTTTTTCTAGGAAGTTCTGTTTTAAGAATGGGTAAGCAGGTGGGCTTTGGCAAAGAGCCAAA [2175]
T23-16_T.europaea           ........-....................T..-.......................................... [2175]
T23-18_T.europaea           ........-....................T..-.......................................... [2175]
```

```
                                                                                                                                                                        *
RCFO01000018_T.occidentalis GAGGAGGTGGTAGGGGCGGAGAAAGCCTTTCATTTCCTTACAAAAGGCACGGTAGGGAAATCCGAGCTTAAGATG [2250]
T23-16_T.europaea           ..............................................................G............ [2250]
T23-18_T.europaea           ..............................................................G............ [2250]

                                                                                                                  *
RCFO01000018_T.occidentalis ATTTTCTGAACTTTCACGTTTCTGCCTTTGGCTGCCTGGAGTTTCGGTTGGTTGGATGGTTGGTTGGTCGGTGGG [2325]
T23-16_T.europaea           ......................C.................................................... [2325]
T23-18_T.europaea           ......................C...............................................T.... [2325]

RCFO01000018_T.occidentalis TGGGTGGGTCTGTCAGTCGGTTAGAACGGTGCCATCATATGCTTCTGCAATGCTCAGCGATGATTTCTGTGCAGG [2400]
T23-16_T.europaea           ...................T.............G......................................... [2400]
T23-18_T.europaea           ...................T.............G......................................... [2400]

RCFO01000018_T.occidentalis GGTGCAGGAACAGAATATCCTGGCCTTCCGGAAAACGTCTTCCTTACTTTGGAGTGACAATCCTGTCTCCAATTT [2475]
T23-16_T.europaea           ....................................A...................................... [2475]
T23-18_T.europaea           ....................................A...................................... [2475]

RCFO01000018_T.occidentalis TCAGTGCGAAACTGGAGGAAAAGGTAGAGAGAGCAGTCAGGAGCGCGTCAAACGACCCATGAATGCATTCATGGT [2550]
T23-16_T.europaea           ......T..............G..................................................... [2550]
T23-18_T.europaea           ......T..............G..................................................... [2550]

RCFO01000018_T.occidentalis TTGGTCTCGAGATCAAAGGCGCAAGGTGGCTCTAGAGAACCCCCACATGCAAAATTCGGAGATCAGCAAGCGTCT [2625]
T23-16_T.europaea           ........................................................................... [2625]
T23-18_T.europaea           ........................................................................... [2625]

RCFO01000018_T.occidentalis GGGATACCAGTGGAAAATGCTTACGGAATCCGAAAAATGGCCATTCTTCGAGGAGGCACAGAGACTACAGGCAGC [2700]
T23-16_T.europaea           ........................................................................... [2700]
T23-18_T.europaea           ........................................................................... [2700]

RCFO01000018_T.occidentalis GCACCGAGAGAAATACCCGGGCTATAAATATCGACCTCGTCTGAAAGGCGGGAAAGCGCGGCAAAGGGACCGGTC [2775]
T23-16_T.europaea           ........................................................................... [2775]
T23-18_T.europaea           ........................................................................... [2775]

RCFO01000018_T.occidentalis GCAGCCCACAGACTCCACTGCCGTGCGGTGCAGCCAGGTGCAAGTGGAGGAGAAGTCGTACGCTTTGGCAAACAG [2850]
T23-16_T.europaea           ..........................T............................C................... [2850]
T23-18_T.europaea           ..........................T............................C................... [2850]

RCFO01000018_T.occidentalis TGATGGCTGTACTACTGTCACACACTCACGAATGGGGCACCAGTTAAAGCACTCGCAGTGCATGAACACAGCCAA [2925]
T23-16_T.europaea           ........................................................................... [2925]
T23-18_T.europaea           ........................................................................... [2925]
```

```
RCFO01000018_T.occidentalis CTAACAGCTGCAACAGGAACCTTTTCACAGCCCCTGCACAAACTTGCCTGAGGATCGGCTGACACCGGCTATGCA [3000]
T23-16_T.europaea           ..................................G........................................ [3000]
T23-18_T.europaea           ..................................G........................................ [3000]

RCFO01000018_T.occidentalis GACCTACCTGTCTATTCCTCTTTAGCATAACTTACAACCCACTTTTTCTCATATTTTCGATGTTCACGTTCTTGA [3075]
T23-16_T.europaea           ........................................................................... [3075]
T23-18_T.europaea           ........................................................................... [3075]

RCFO01000018_T.occidentalis GCTAACAAAGACTTCATTCGTCTCGACTTTACTATGACTTCAGCTTTAAGTTAACTTCGGAAACAGAGTGTAAAC [3150]
T23-16_T.europaea           ........................................................................... [3150]
T23-18_T.europaea           ........................................................................... [3150]

RCFO01000018_T.occidentalis GTCCTTGGGGTGTTTAGCAATAACGACATTCTGAAGTGTCTAACTGGTGTTGAGGTAGCATAAGGGAGAACTGAC [3225]
T23-16_T.europaea           ............................................C.............................. [3225]
T23-18_T.europaea           ............................................C.............................. [3225]

RCFO01000018_T.occidentalis TTGATAAGAAAGTATGCACACTGTGCAGGTAGAAAAACAGTGGCACTGAGCTGAAAACTCTGCAGCCCCTAAGCA [3300]
T23-16_T.europaea           ...........................................................T............... [3300]
T23-18_T.europaea           ...........................................................T............... [3300]

RCFO01000018_T.occidentalis ATCCCTAAGAAATCTTCCC---TTTGATCTTTCAGCTGCCTTGCATGTCTCTGTAAACCTGGCAATTAACCAATG [3375]
T23-16_T.europaea           ...................TTC.................T............A...................... [3375]
T23-18_T.europaea           ...................TTC.................T............A...................... [3375]

RCFO01000018_T.occidentalis GGCATGTTAATTGCATCTTCTTTACTAGAGTTTACTGTTCCTACTCCCACCTGTGGAGAAACCGGTTTGGCAACC [3450]
T23-16_T.europaea           ...............................AA.........................................G [3450]
T23-18_T.europaea           ...............................AA.........................................G [3450]

RCFO01000018_T.occidentalis CAATTCCTCCACTCCACACGAAATTTCCCAGCTTTGACCTCTGTTTATGTCCAACCCAACGTAACTCCAAAGTGG [3525]
T23-16_T.europaea           ................................................A...........A.............. [3525]
T23-18_T.europaea           ................................................A...........A.............. [3525]

                                                     *
RCFO01000018_T.occidentalis TAAAGTGAACTTCTATTTTACTCCCGTTCATTTTCATTTCTGTAATGATGCCACGGTTTTGCCTTTTTGTAAAAAAAAAAGC [3607]
T23-16_T.europaea           ..................A......R............................................poly-A       [3607]
T23-18_T.europaea           ..................A...................................................poly-A       [3607]
```

Alignment S3,b. Alignment of nucleotide sequences of the *Sry* gene second exon fragment (158 bp) of several species of the Talpidae family (*Talpa occidentalis*, *T. europaea*, and *T. romana*), the Soricidae family (*Neomys anomalus* and *Crocidura suaveolens*), and the Erinaceidae family (*Erinaceus algirus*).

```
RCFO01000018_T.occidentalis TCATGGTTTGGTCTCGAGATCAAAGGCGCAAGGTGGCTCTAGAGAACCCCCACATGCAAAATTCGGAGATCAGCA [ 75]
X90843_T.occidentalis       ........................................................................... [ 75]
X90863_T.romana             ........................................................................... [ 75]
X95595_T.europaea           ........................................................................... [ 75]
T23-16_T.europaea           ........................................................................... [ 75]
T23-18_T.europaea           ........................................................................... [ 75]
X90864_N.anomalus           ........................................................................... [ 75]
X90865_C.suaveolens         ........................................................................... [ 75]
X90866_E.algirus            .......G.................A.....A.....C........T..A..A........CA.A.......... [ 75]

RCFO01000018_T.occidentalis AGCGTCTGGGATACCAGTGGAAAATGCTTACGGAATCCGAAAAATGGCCATTCTTCGAGGAGGCACAGAGACTAC [150]
X90843_T.occidentalis       ........................................................................... [150]
X90863_T.romana             ........................................................................... [150]
X95595_T.europaea           ........................................................................... [150]
T23-16_T.europaea           ........................................................................... [150]
T23-18_T.europaea           ........................................................................... [150]
X90864_N.anomalus           ........................................................................... [150]
X90865_C.suaveolens         ........................................................................... [150]
X90866_E.algirus            ...TG.....T............C.......T...G.A......CA...T......C.............GT... [150]

RCFO01000018_T.occidentalis AGGCAGCG [158]
X90843_T.occidentalis       ........ [158]
X90863_T.romana             ........ [158]
X95595_T.europaea           ........ [158]
T23-16_T.europaea           ........ [158]
T23-18_T.europaea           ........ [158]
X90864_N.anomalus           ........ [158]
X90865_C.suaveolens         ........ [158]
X90866_E.algirus            .A....T. [158]
```
